# Supplementary material for: A metagenomic catalog of the early-life human gut virome
Source: Nat Commun. 2024 Feb 29;15:1864. doi: 10.1038/s41467-024-45793-z (PMC10904392; doi:10.1038/s41467-024-45793-z)
Supplement: Supplementary file 1 — Supplementary Information [file 41467_2024_45793_MOESM1_ESM.pdf]

Supplementary Information: A metagenomic catalog of the early-life human gut virome

S. Zeng et al.

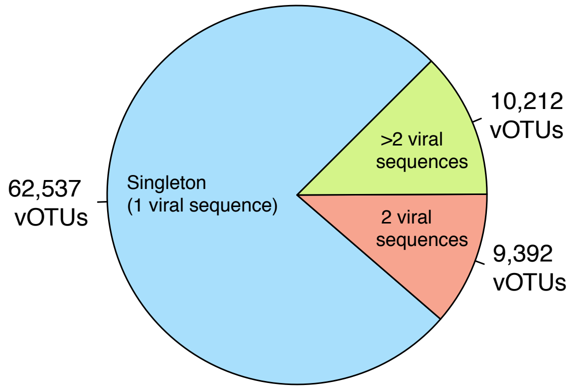

Supplementary Fig. 1 Categories of 82,141 vOTUs from the ELGV catalog. The proportion of vOTUs with single, two, and > 2 viral sequences.

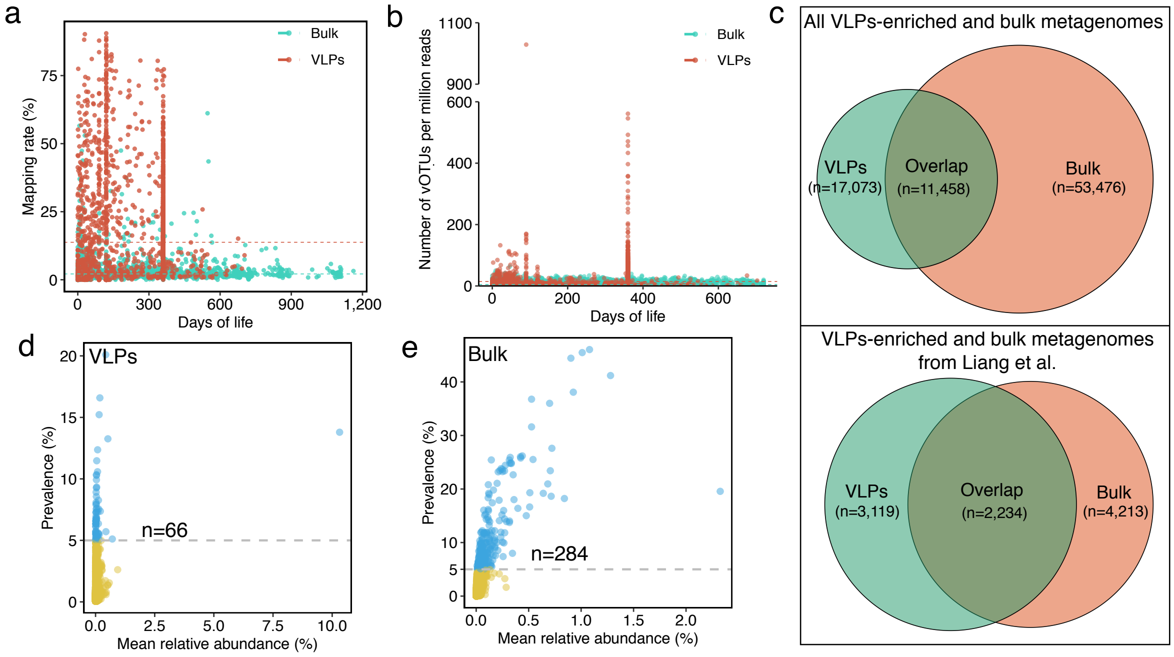

Supplementary Fig. 2 a, The mapping rate of all 1,865 VLPs-enriched and 6,265 bulk metagenomes as a function of the age of infants. **b**, Number of vOTUs per million sequenced reads of all 1,865 VLPs-enriched and 6,265 bulk metagenomes as a function of the age of infants. **c**, The number of vOTUs that were overlapped by VLPs and bulk based on the large combined fecal samples (top) or the same fecal samples that were subjected to both VLPs-enriched and bulk sequencing from Liang et al (bottom). **d-e**, The prevalence and mean relative abundance of 28,531 and 64,934 vOTUs respectively in 1,682 VLPs-enriched (**d**) and 6,205 bulk (**e**) metagenomes that were detected with at least one vOTU representative. A number of 66 and 284 vOTUs were populated among ≥ 5% (indicated by the horizontal line) of VLPs-enriched and bulk metagenomes, respectively.

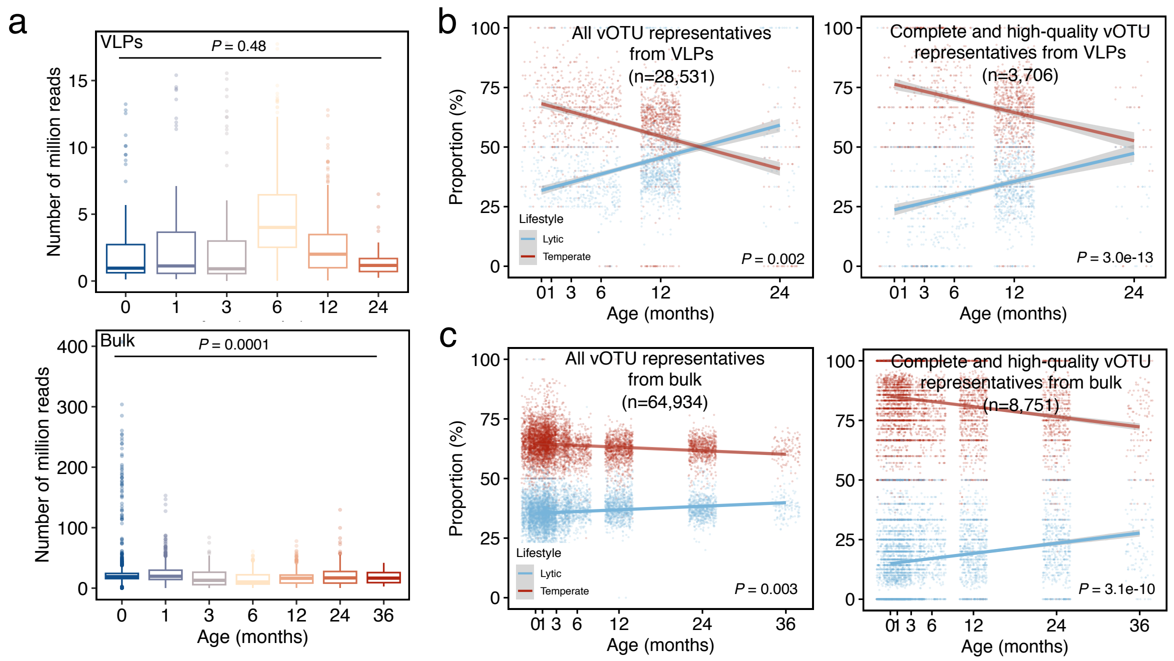

Supplementary Fig. 3 The sequencing depth and proportion of temperate and lytic viruses in the human gut virome in the first three years of life. a, The sequencing depth of infant gut VLPs-enriched (top) and bulk (bottom) metagenomes distributed throughout age. The *P* values were obtained by linear mixed modelling with 'study' as random factor. **b-c**, The proportion of temperate and lytic viruses as a function of infant age, analyzed with all vOTU representatives or only based on complete and high-quality vOTU representatives in VLPs-enriched or bulk metagenomes. The *P* values were obtained by linear mixed modelling with 'study' as random factor. Shaded area indicates the estimated 95% confidence interval.

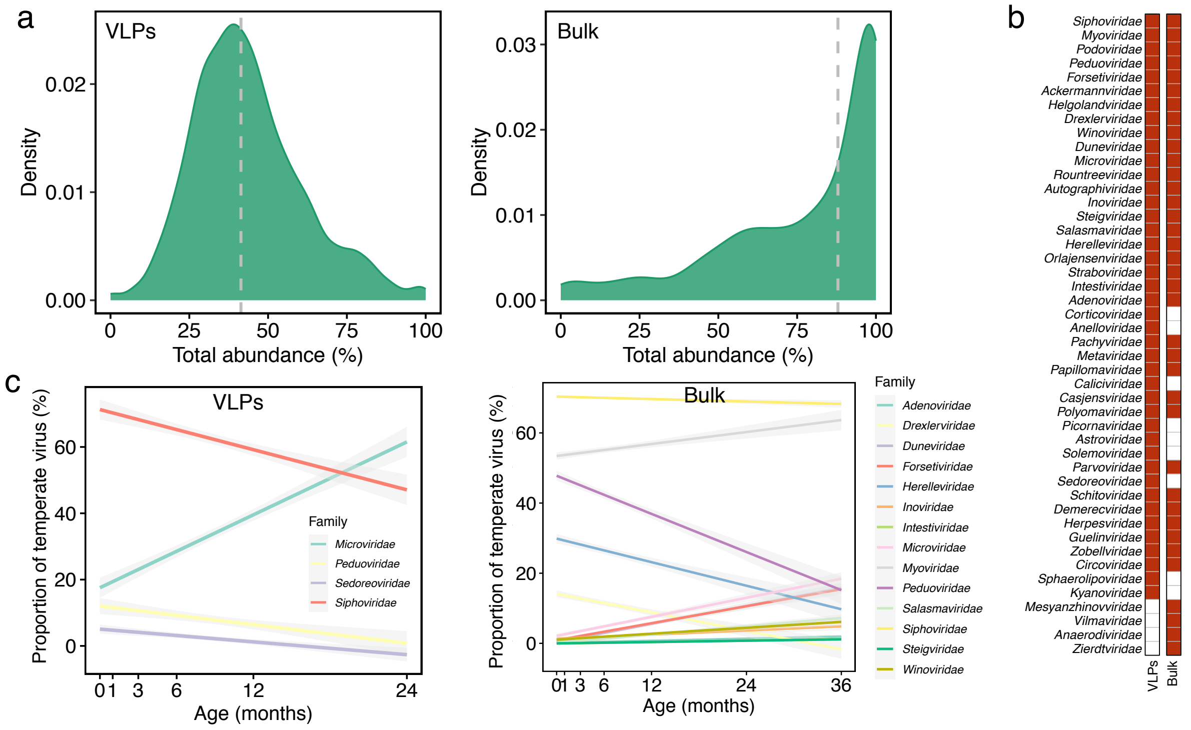

Supplementary Fig. 4 The distribution of relative abundance and the proportion of temperate viruses at the family level. a, The total 42 classified viral families from 1,682 VLPs-enriched metagenomes accounted for a median relative abundance of 87.9% (the vertical line; left), while 37 viral families from 6,205 bulk metagenomes accounted for a median relative abundance of 41.4% (the vertical line; right). **b**, The presence of viral families that were detected in VLPs-enriched and bulk metagenomes. **c**, The changes of temperate viruses from each families (linear regression, $P < 0.05$) based on VLPs-enriched (left) and bulk (right) metagenomes. Shaded area indicates the estimated 95% confidence interval.

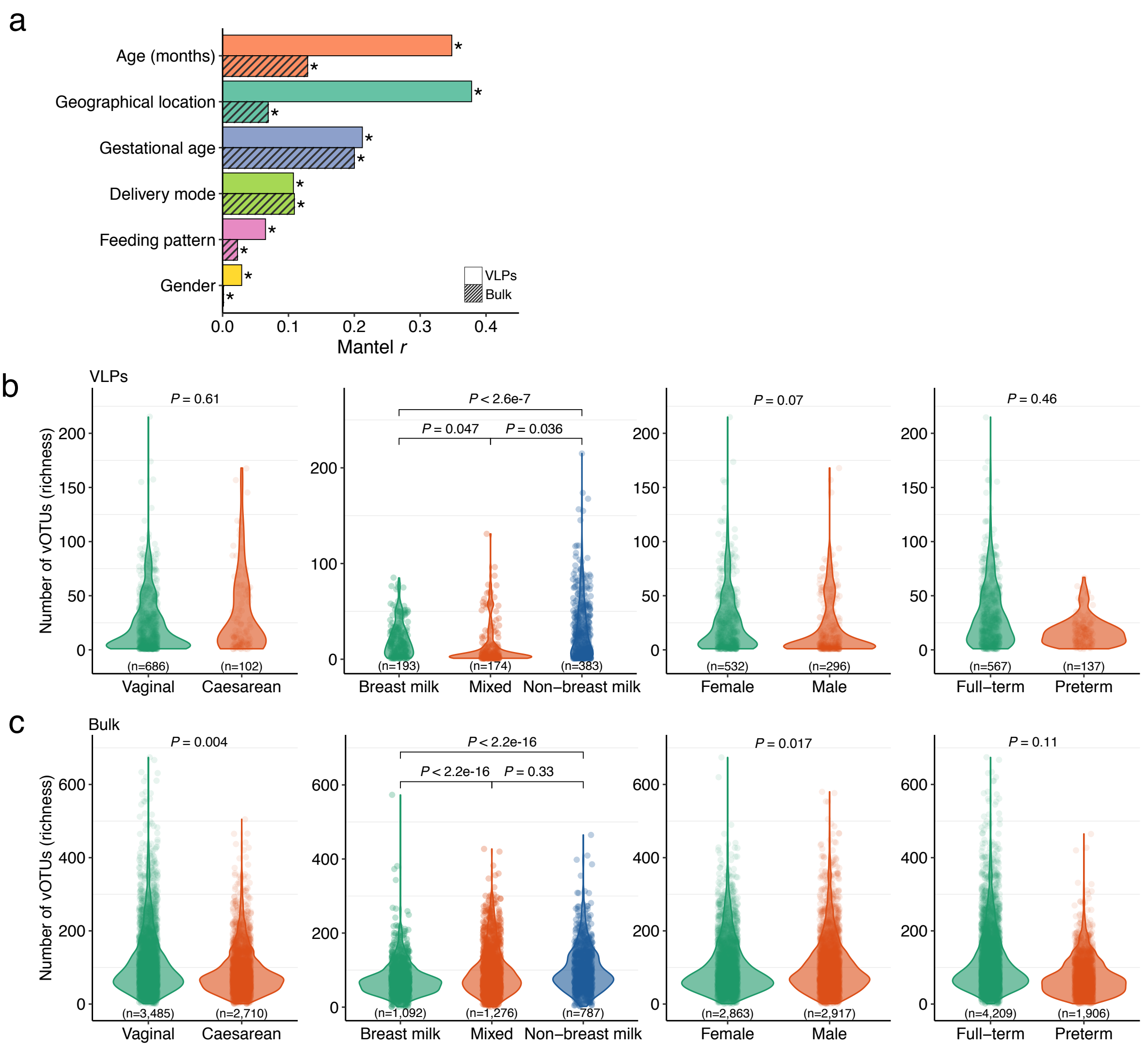

Supplementary Fig. 5 Influences of factors on the development of the early-life human gut virome. a, Effect size (R^2) explained by clinical factors as determined by MANTEL based on VLPs-enriched or bulk metagenomes. Asterisk (*) denotes the significance (FDR < 0.05) of each factor. **b-c**, Influences of delivery mode, feeding pattern, gestational age, and gender on the alpha diversity of the early-life human gut virome based on VLPs-enriched (**b**) or bulk metagenomes (**c**). The *P* values were obtained by two-sided Wilcoxon test blocked by ‘study’.

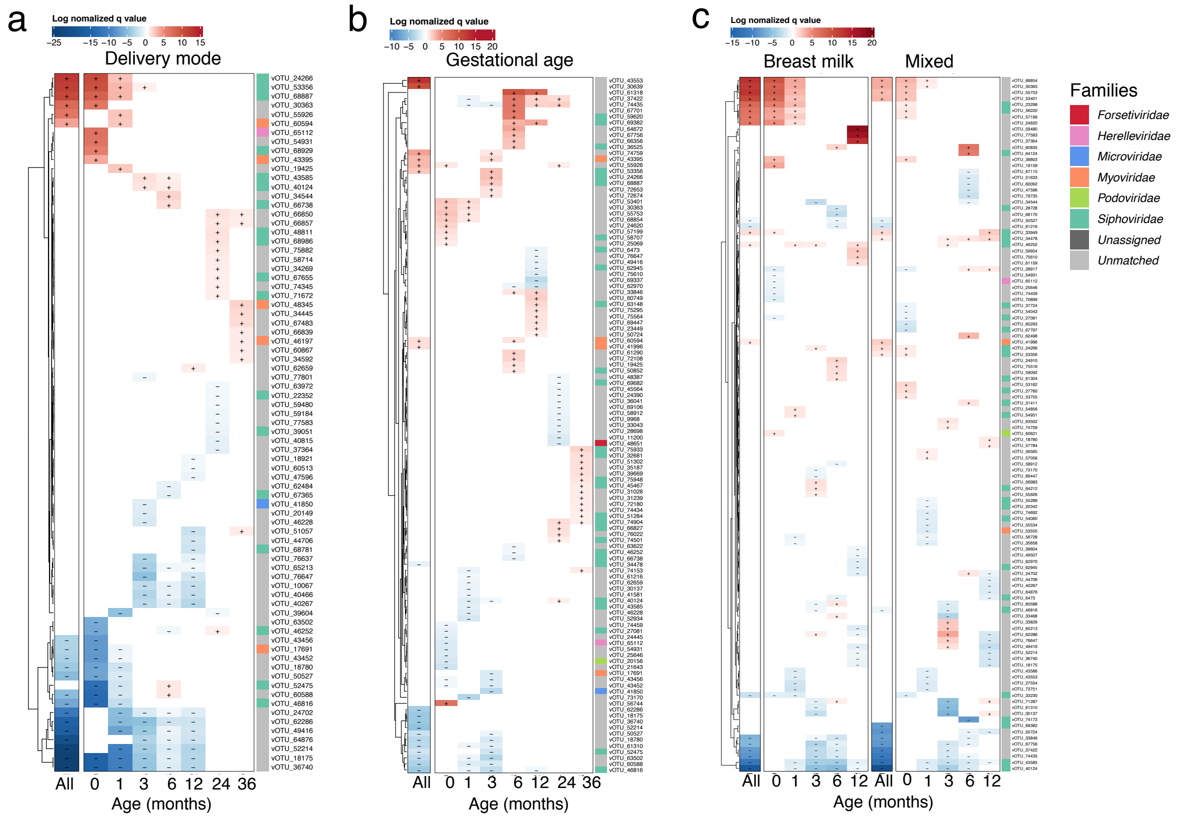

Supplementary Fig. 6 The vOTUs associated with the clinical factors. a-c, The vOTUs significantly (MaAsLin2 with 'subjects' as random effect and other metadata factors as fixed effects; $q < 0.25$) associated delivery mode taking vaginal delivery as reference (**a**), gestational age taking infants born full-term as reference (**b**), and feeding pattern taking non-breast milk feeding as reference (**c**), which were analyzed with all samples together or stratified by infant age (months). Only the top 20 significant vOTUs ($q < 0.25$) from outputs by MaAsLin2 with all samples together or stratified by infant age (months) are plotted. The sign (+) indicates positive and (–) negative associations with infants born by C-section (**a**), preterm (**b**), and fed by breast milk exclusively or partially (**c**), respectively. The stratified analysis of feeding pattern for infants at month 24 and 36 was not available due to insufficient factor levels. The color gradient indicates the strength of the association calculated by $-log_{10}(q\text{-}value) \times sign(coeff)$.

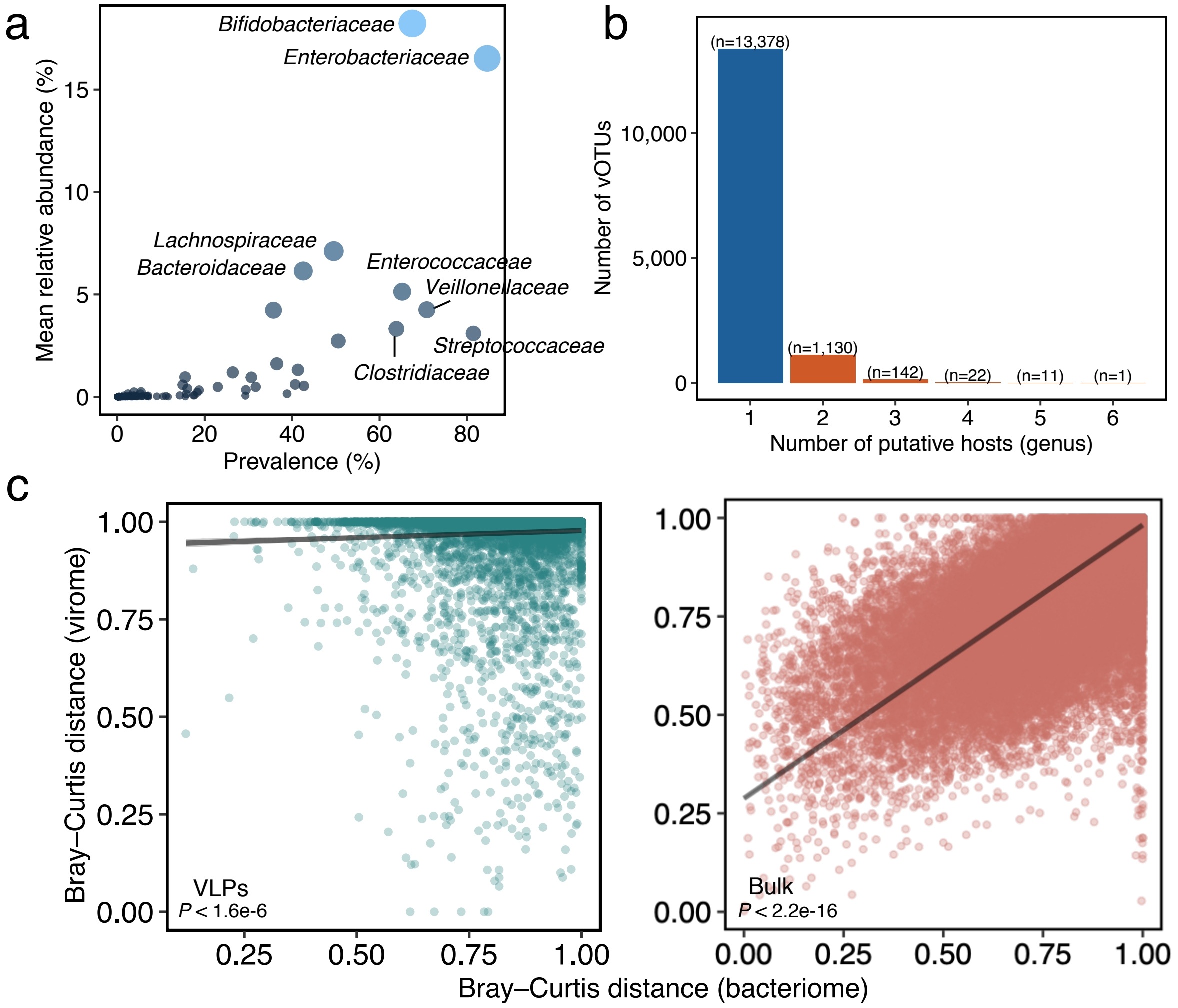

Supplementary Fig. 7 Description of the early-life human gut bacteriome, virome and their interactions. a, The most abundant bacterial families detected in the 6,265 bulk metagenomes early in life. **b**, Number of predicted putative hosts at the genus level for each vOTU. **c**, Correlation between the gut virome and bacteriome regarding the beta diversity based on Bray–Curtis distances of viral and bacterial species with a prevalence ≥ 1% for VLPs-enriched (left) or ≥ 5% for bulk metagenomes (right). Only randomly selected 1% of all Bray–Curtis distances between from bulk metagenomes (right) were plotted. The *P* values were obtained from linear regression, and shaded area indicates the estimated 95% confidence interval.

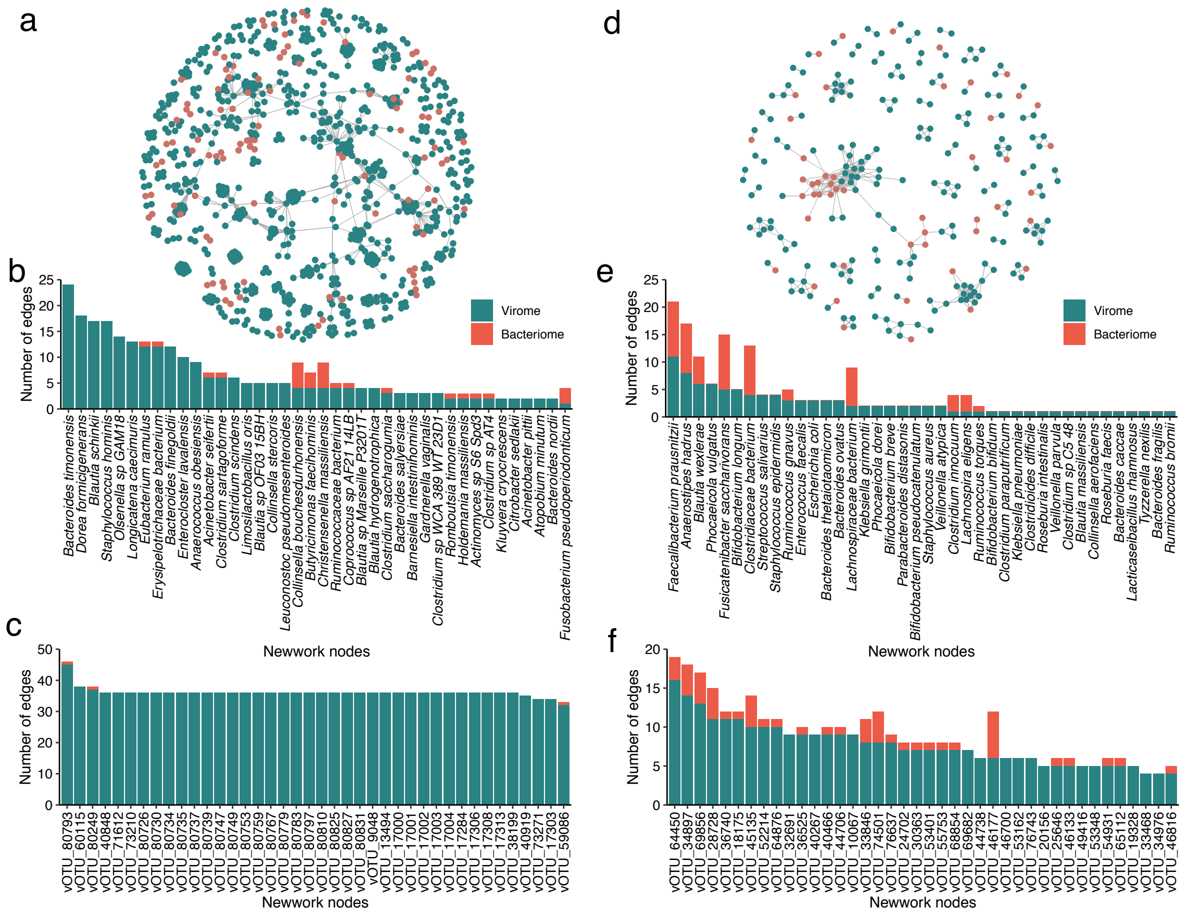

Supplementary Fig. 8 Co-occurrence networks at the species level for VLPs-enriched and bulk metagenomes. **a**, Co-occurrence networks of 1,031 vOTUs and 356 bacteria at the species level with ≥ 1% prevalence among 141 samples that were processed by using both VLPs and bulk metagenomic sequencing. A positive connection is indicated in a grey edge (Spearman's ρ ≥ 0.6; FDR < 0.05). **b**, The top 40 bacterial species with the highest number of connections based on co-occurrence network of (**a**). **c**, The top 40 vOTUs with the highest number of connections based on co-occurrence network of (**a**). **d**, Co-occurrence networks of 283 vOTUs and 159 bacteria at the species level with ≥ 5% prevalence among 6,066 bulk metagenomes. A positive connection is indicated in a grey edge (Spearman's ρ ≥ 0.6; FDR < 0.05). **e**, The top 40 bacterial species with the highest number of connections based on co-occurrence network of (**d**). **f**, The top 40 vOTUs with the highest number of connections based on co-occurrence network of (**d**).

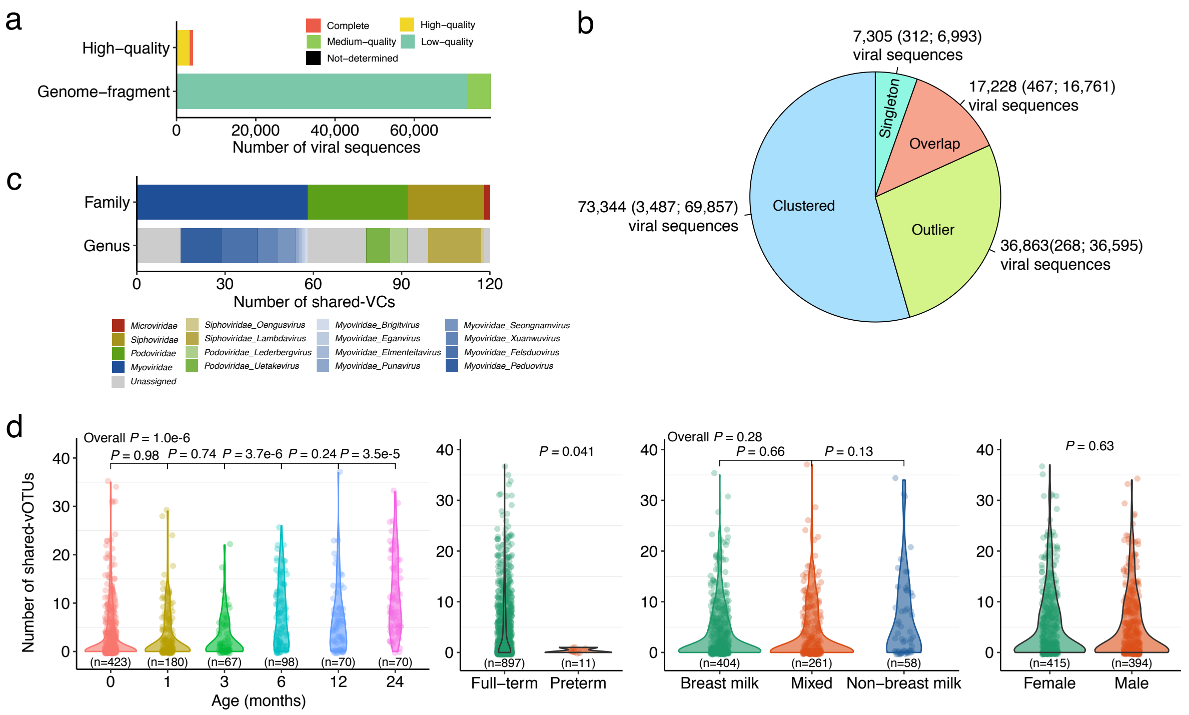

Supplementary Fig. 9 The shared virome between mother and their infants. a, The quality of 83,543 maternal viral sequences based on CheckV. The bar shows the proportion of viral sequences with different qualities from CheckV stratified by the standard of MIUViG (high-quality vs. genome-fragment). **b**, The proportion of categories of viral clusters (VCs) from using vConTACT2 with all 130,206 viral sequences of bulk metagenomes from mother-infant dyads with NCBI Viral RefSeq version 211. The numbers in parenthesis indicate the number of viral sequences from RefSeq and mother-infant dyads, respectively. The category "Clustered/Singleton" from vConTACT2 was regrouped into "Singleton" category. **c**, The taxonomic assignment of the paired mother-infant shared-VCs based on bulk metagenomes. **d**, Influences of the clinical factors on the shared-vOTUs between paired mother-infant dyads based on bulk metagenomes. The *P* values were examined using two-sided Wilcoxon test blocked by 'study' for two-level comparisons and Kruskal–Wallis test blocked by 'study' for three or more-levels comparisons.

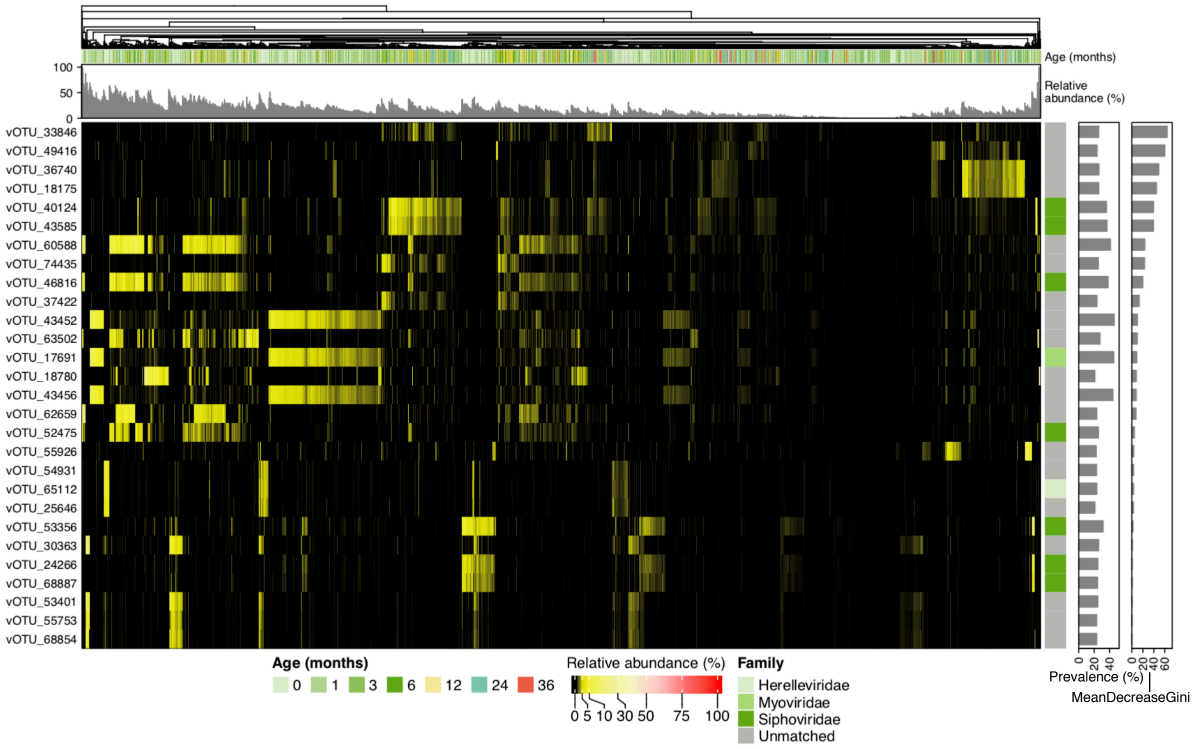

Supplementary Fig. 10 The relative abundance of 28 vOTUs in the early-life human gut metagenomes. The rows represent the vOTUs, and the columns represent the early-life human gut bulk metagenomes. The column annotations indicate the infant age (months) and total relative abundance (from top to bottom) in each bulk metagenome, respectively. The row annotations indicate the taxonomic assignment of vOTUs at the family level, the prevalence (%) across all bulk metagenomes, and the value of MeanDecreaseGini in the random forest classifier model (from left to right), respectively.
