# Supplementary material for: A metagenomic catalog of the early-life human gut virome
Source: Nat Commun. 2024 Feb 29;15:1864. doi: 10.1038/s41467-024-45793-z (PMC10904392; doi:10.1038/s41467-024-45793-z)
Supplement: Supplementary file 3 — Description of Additional Supplementary Files [file 41467_2024_45793_MOESM3_ESM.pdf]

Supplementary Data legends

Supplementary Data 1

Metadata of 8,130 fecal metagenomes from infants across 35 studies, including 6,265 bulk and 1,865 VLPs-enriched metagenomes.

Supplementary Data 2

The quality of the ELGV catalog with 160,478 viral sequences assessed by CheckV.

Supplementary Data 3

The description of 82,141 vOTUs, including the quality, vOTU number, taxonomic annotation, and lifestyle.

Supplementary Data 4

The comparison, dynamics and statistics of 42 and 37 individual viral families (mean relative abundance, %) from VLPs-enriched and bulk metagenomes, respectively, in the first three years of life stratified into infant age of months 0, 1, 3, 6, 12, 24, and 36.

Supplementary Data 5

Differential vOTUs from VLPs-enriched or bulk metagenomes influenced by the clinical factors, including delivery mode, gestational age, and feeding pattern, which were analyzed with MaAsLin2 for significance. The *P* values were corrected with default settings, which were reported as *q* values.

Supplementary Data 6

The number of connections between bacterial and viral species based on co-occurrence network from VLPs-enriched or bulk metagenomes.

Supplementary Data 7

Metadata of 373 paired mother-infant dyads including 460 maternal fecal bulk metagenomes and 1,000 infant fecal bulk metagenomes; metadata of 26 paired mother-infant dyads including 26 maternal fecal VLPs-enriched metagenomes and 48 infant fecal VLPs-enriched metagenomes. Infants with subject ID 115T from study WampachL_2018, and A00106T, C02223T, and C02294T from study ShaoY_2019 are twins of infants with subject ID 115 from WampachL_2018, and A00106, C02223, and C02294 from study ShaoY_2019.

Supplementary Data 8

A number of 1,669 vOTUs were shared by the paired mother-infant dyads, including 5,490 shared events from 329 paired mother-infant dyads.

Supplementary Data 9

Comparisons of the ELGV catalog (n = 82,141) to other databases, including CHVD (n = 40,203), GPD (n = 124,775), GVD (n = 16,379), and MGV (n = 46,464) and isolated viruses in RefSeq (n = 8,879).

Supplementary Data 10

The relative abundance (%) of the ELGV viral representatives in 521 VLPs-enriched and 510 gut metagenomes from adults.

Supplementary Data 11

The relative abundance (%) of the ELGV viral representatives in 302 gut bulk metagenomes from infants in the first year of life.

Supplementary Data 12

The functional annotations of 407 and 28 vOTU representatives from VLPs-enriched and bulk, respectively, against KEGG, COG, and Pfam databases.
